# circTADA2A inhibited SLC38A1 expression and suppresses melanoma progression through the prevention of CNBP trans-activation

**DOI:** 10.1371/journal.pone.0301356

**Published:** 2024-04-18

**Authors:** Longjun Zhang, Le Zhang, Chi Zhang, Sunan Shi, Zhilei Cao, Changliang Shao, Jie Li, Yingshun Yang, Xi Zhang, Jian Wang, Xiangyun Li

**Affiliations:** 1 Department of Plastic Surgery, Affiliated Xing Tai People Hospital of Hebei Medical University, Xingtai, Hebei, China; 2 Department of Oral Surgery, Hebei Provincial Eye Hospital, Xingtai, Hebei, China; 3 Department of Cataract, Hebei Provincial Eye Hospital, Xingtai, Hebei, China; 4 Department of Otolaryngology, Hebei Provincial Eye Hospital, Xingtai, Hebei, China; 5 Department of Operation and Anaesthesia, Hebei Provincial Eye Hospital, Xingtai, Hebei, China; 6 Department of Optometry, Hebei Provincial Eye Hospital, Xingtai, Hebei, China; University of South Florida, UNITED STATES

## Abstract

**Background:**

CircTADA2A has been demonstrated to play critical roles in the occurrence and development of human cancer. However, the expression pattern and biological mechanisms of circTADA2A in melanoma remains largely unknown.

**Methods:**

CircTADA2A were detected by quantitative real-time RT-PCR (qRT-PCR) and validated by Sanger sequencing. Function of circTADA2A and its protein partner in melanoma cells was investigated using RNA interference and overexpression assays. Interaction of circTADA2A, CCHC-type zinc finger nucleic acid binding protein (CNBP) and solute carrier family 38 member 1 (SLC38A1) was confirmed by RNA immunoprecipitation, RNA pull-down, and dual-luciferase reporter assay. The expression of genes and proteins were detected by qRT-PCR and western blot assays.

**Results:**

Data from the investigation showed that a novel circRNA (circTADA2A, hsa_circ_0043278) was markedly downregulated in melanoma cells. Functionally, circTADA2A repressed cell proliferation, migration, invasion in melanoma cells. Mechanistically, circTADA2A interacted with CNBP, acting to suppress the binding of CNBP to the SLC38A1 promoter and subsequently restrained SLC38A1 transcription, which resulting in repression of melanoma progression.

**Conclusions:**

CircTADA2A suppresses melanoma progression by regulating CNBP/SLC38A1 axis, indicating a potential therapeutic target in melanoma.

## Introduction

Melanoma is the most common lethal cutaneous malignant tumors originated from melanocytes [1]. Unfortunately, the incidence of melanoma has been rapidly increasing worldwide [1, 2]. In spite of advancement in therapeutic strategies, but the prognosis of melanoma remains dismal due to chemotherapy resistance and metastasis [3, 4]. Thus, further research is urgently needed to developing new effective therapeutic targets for melanoma.

Circular RNAs (circRNAs) are a type of endogenous noncoding RNAs (ncRNAs) that lack 5′and 3′ends and poly (A) tails, and thus exist as continuous loop RNAs [5]. Due to the particular feature, circRNAs have the unique property of being highly tolerance to exonuclease digestion compared with other linear RNAs [5, 6]. In recent years, accumulating investigations have found that circRNAs are emerging as critical regulators in carcinogenesis and cancer progression by competitively sponging regulatory miRNAs or interacting with RNA binding proteins (RBPs) [6, 7]. For instance, in osteosarcoma, circPVT1 promotes cell invasion and metastasis by acting as a miR-205-5p sponge [8]. As a nuclear down-regulated noncoding RNA, circHuR suppresses HuR expression and gastric cancer progression by inactivating CNBP [9]. CircCLK3 is known to promote the growth and metastasis of cervical cancer by sponging miR-320a and enhancing the expression of FoxM1 [10]. However, the biological functions and underlying mechanisms of circRNAs in melanoma progression remain largely elusive.

CircTADA2A, derived from exons 5 and 6 of the transcriptional adaptor 2A (TADA2A) gene, has been implicated in various pathological processes of human cancers [11, 12]. CircTADA2A has been reported to promote cell proliferation, migration, and invasion in osteosarcoma [11] and non‑small cell lung cancer [12]. Whereas, circTADA2A was identified as a tumor suppressor in colorectal and breast cancer [13, 14]. Therefore, the role of circTADA2A in cancer is controversial, and its functional role in melanoma remains unclear.

In the present study, we identified hsa_circ_0043278 (circTADA2A) that acts as a tumor suppressor in melanoma. The expression of circTADA2A is significantly downregulated in melanoma cell lines and is positively associated with melanoma progression by interacting with CCHC-type zinc finger nucleic acid binding protein (CNBP) to influence the expression of solute carrier family 38 member 1 (SLC38A1). CircTADA2A may exert regulatory functions and serve as a target for melanoma treatment.

## Methods

### Bioinformatics analysis

A thorough search for available melanoma microarray datasets was performed with the Gene Expression Omnibus (GEO) database (accession numbers: GSE31909 and GSE35388) based on dataset relevance, data availability, dataset selection criteria, data quality assessment and data heterogeneity. Differentially expressed genes (DEGs) were screened with R software with the threshold set as log2 |fold change| ≥ 2 and pvalue < 0.05. The analysis results were overlapped to investigate the downstream genes. In addition, The Encyclopedia of RNA Interactomes (ENCORI) database (http://starbase.sysu.edu.cn/index.php) was used to predict the binding molecules of CNBP (clusterNum > = 30).

### Cell culture and transfection

The human melanoma cells (A375 and A2058) and normal human melanocytes (HeMa-Lp) were purchased from the American Type Culture Collection (ATCC). All the cells were cultured in high-glucose Dulbecco’s Modified Eagle Medium (DMEM, GibcoBRL, Invitrogen, Carlsbad, CA, USA) and supplemented with a 10% fetal bovine serum (FBS; Thermo Fisher Scientific, Waltham, MA, USA), and 1% penicillin–streptomycin. During the culture period, Cells were grown at 37°C in a humid incubator with 5% CO_2_.

The melanoma cells were seeded and incubated for 24 h, and then transfected with oligonucleotides or plasmids using Lipofectamine 3000 (Invitrogen, Carlsbad, CA, USA). Briefly, pcDNA or small interfering RNA (siRNA) was diluted in serum-free medium, and mixed with Lipofectamine 3000 accordance with the manufacturer’s instructions. After incubation for 6–8 h, the cellular supernatant was removed and cultured with fresh complete medium for 48 h.

### Quantitative real-time polymerase chain reaction (qRT-PCR)

The cultured cells were extracted using Trizol reagent (Invitrogen, CA, USA) according to the manufacturer’s protocols. Real-time PCR analyses were performed using an ABI 7300 Fast RealTime PCR System (Applied Biosystems, Foster City, CA) and SYBR Green PCR kit (Applied TaKaRa, Otsu, Shiga, Japan). Obtained data were analyzed with the 2^-ΔΔCt^ method, and U6 and β-actin acted as references. The specific primers are listed in the **Table 1**.

**Table 1:** The sequence of PCR primers.

Primer	Sequence
TADA2A U6	Forward: 5’ GGACAGCAGGCATTACCAA 3’ Reverse: 5’ AGGGACCAACCTCACCATC 3’ Forward: 5’ TGCGGGTGCTCGCTTCGGCAGC 3’ Reverse: 5’ -CCAGTGCAGGGTCCGAGGT 3’
FMNL2 GAPDH FMN2 SLC38A1 AUTS2 SATB2	Forward: 5’ GGAGCTGGATGTCGTTCGG 3’ Reverse: 5’ AGGCAGTGGTGGTGGAGGAG 3’ Forward: 5’ CAAGTATGATGACATCAAGAAGGTGG 3′ Reverse: 5’ GGAAGAGTGGGAGTTGCTGTTG 3′ Forward: 5’ CAGGACAAAGGGAGTAGGA 3’ Reverse: 5’ GTCTCCAGGTCAACCACAG 3’ Forward: 5’ ACCTAAGCAACGCCATTAT 3’ Reverse: 5’ GGCAGAGGGTAGTTCATTT 3’ Forward: 5’ TCTTCCATTCCTATCCTCCTG 3’ Reverse: 5’ CTGTCAACCTCGGGTCCTT 3’ Forward: 5’ AGGAGTTTGGGAGATGGTA 3’ Reverse: 5’ TTGGTTTCGGATTGGAGTA 3’

### RNase R treatment

RNase R digestion reaction as performed as previously reported [15]. Total RNA (5 μg) was incubated for 15 min at 37°C with or without 3 U/μg RNase R (Epicentre Biotechnologies, Madison, WI, USA). The RNA was subsequently subjected to qRT-PCR.

### RNA fluorescent in situ hybridization (FISH)

For FISH, the seeded cells were fixed, permeabilized, and prehybridized. Subsequently, hybridization was carried out with a FISH probe in a moist chamber at 37°C in the dark overnight. FISH kit and specific probes targeting circTADA2A were purchased from Guangzhou RiboBio Co., Ltd. (China). Then, the cells were counterstained with DAPI. Images were acquired by fluorescence microscopy (Olympus, Tokyo, Japan) and analyzed with ImageJ.

### Cell Counting Kit-8 (CCK-8) assay

Cell viability in different groups was measured using CCK-8 assays (Dojindo, Japan) according to the manufacturer’s protocol. Briefly, cells were seeded in 96-well plates at a density of 4×10^3^ cells per well. The absorbance at 450 nm was measured using a microplate reader (Thermo, USA).

### Colony formation assay

Transfected cells or stable cells were seeded into 6-well plate (5×10^2^/well). The cultures were maintained at 37°C in a 5%CO_2_ incubator for 2 weeks. The colonies were fixed with 4% paraformaldehyde and stained with 0.5% crystal violet. The numbers of the colony were counted and photographed using an inverted microscope (Olympus IX71, Japan).

### 5‑ethynyl‑20‑deoxyuridine (EdU) analysis

Cell proliferation ability was detected using the EdU assay kit (Ribobio, Guangzhou, China). Briefly, melanoma cells were seeded into 96-well plates (1 × 10^4^cells/well) in triplicate. After cultured for 24h, the cells were incubated with 50μM EdU for 2h. Subsequent staining and visualization were carried out in accordance with the manufacturer’s instructions. The ratio of EdU-positive cells among total cells was calculated and analyzed in three random fields.

### Transwell cell migration and invasion assay

The migration and invasion abilities of melanoma cells were assayed using Transwell inserts and Matrigel-coated Transwell (Corning, MA, USA). Briefly, 2 × 10^4^ melanoma cells were suspended in 200 μL of medium free of serum, and added into the top chamber. 500 mL culture medium containing 10% FBS was added into the lower chamber. 24 h after incubation, a cotton swab was used to remove cells on the upper surface of the membrane gently and the cells were fixed in 4% paraformaldehyde, then 0.5% crystal violet was used to stain these cells for 20 min. The migrating or invading cells were photographed and counted under a microscope.

### Wound healing assay

Wound healing assay was utilized to evaluate the ability of cell migration. We seeded 3 × 10^5^ cells into 6‐well plates in triplicate. After cells grew to about 90% confluent, the scratch at the center of wells was made with sterile 200μl pipe tips. Cells were washed, cultured and photographed with a phase-contrast microscope. Calculation of Cell Migration Rate: Divide the change in wound width by the initial wound width, and then multiply by 100 to obtain the percentage of cell migration rate. Cell Migration Rate (%) = (Change in Wound Width / Initial Wound Width) * 100.

### Dual-luciferase reporter assay

The promotors of SLC38A1 that contained the wild type (WT) or mutant type (MT) binding sites of CNBP were all synthesized by Ruibiotech (Beijing, China). Then we cloned the WT or MT sequences of SLC38A1 into pmirGLO (Promega, USA). After that, co-transfections were conducted with the corresponding plasmids and overexpression vector/overexpression-NC or shRNA/ shRNA-NC of CNBP in melanoma cells respectively. After 48 h of transfection, the relative luciferase activity was conducted using Dual Luciferase Reporter Assay System (Promega, WI, USA).

### Cell cycle analysis by flow cytometry

For cell cycle assay, the melanoma cells were stained with propidium iodide by the cycletest plus DNA reagent kit (BD Biosciences, USA) based on the manufacturer’s manual. The cell cycle was determined via flow cytometry and analyzed by Cell Quest Modfit software.

### RNA pull-down assay

Following the manufacturer’s instructions of RNA pull-down kit (Bersinbio, China), the cells were harvested and lysed in lysis buffer. Then, the streptavidin-coated magnetic beads (Invitrogen) were incubated with the biotin-labeled circTADA2A or Ctrl probes at 30°C overnight. We incubated the cell lysates with RNA probe-coupled beads, and collected the proteins pulled down using the elution buffer. Eventually, the retrieved proteins were further identified or validated by western blot.

### RNA immunoprecipitation (RIP) assay

RIP was conducted with the Magna RIP RNA-Binding Protein Immunoprecipitation Kit (Millipore, USA) according to the manufacturer’s instructions. Briefly, magnetic beads coated with 5 μg of specific antibodies were incubated with prepared cell lysates overnight at 4°C. Then, the RNA-protein complexes were washed 6 times and incubated with proteinase K digestion buffer. RNA was finally extracted by phenol-chloroform RNA extraction methods. The relative expression of RNA was determined by qRT-PCR and normalized to the input.

### Western blot assay

The total protein lysate was extracted with RIPA (Beyotime, China), and determined with BCA Protein Assay Kit (KeyGEN, China). The protein with equal amount was separated by 6–12% SDS-PAGE, and transferred on PVDF membranes. Following blocking, we incubated the membranes with primary antibody at 4°C overnight, then with secondary antibody at room temperature for 2h. Specific primary antibodies used were as follow: anti-CNBP (67109-1-Ig, Proteintech, China), anti-SLC38A1 (12039-1-AP, Proteintech, China), anti-PCNA (10205-2-AP, Proteintech, China), anti-MMP-9 (10375-2-AP, Proteintech, China), anti-GAPDH (60004-1-Ig, Proteintech, China). After incubating with the fluorescein-conjugated secondary antibody, the signals were detected using ECL chemiluminescence reagent (Millipore, USA) and a chemiluminescence system (Bio-Rad, USA) and analyzed using Image Lab Software.

### Statistical analysis

All data in this study was represented at least three experiments and they are expressed as the mean ± SEM. Differences between groups were compared using Student’s t-test or Two Way ANOVA. Statistical significance was determined as P<0.05. The analysis was performed using GraphPad Prism software (San Diego, CA, USA).

## Results

### CircTADA2A is downregulated in melanoma cells

**Fig 1:**
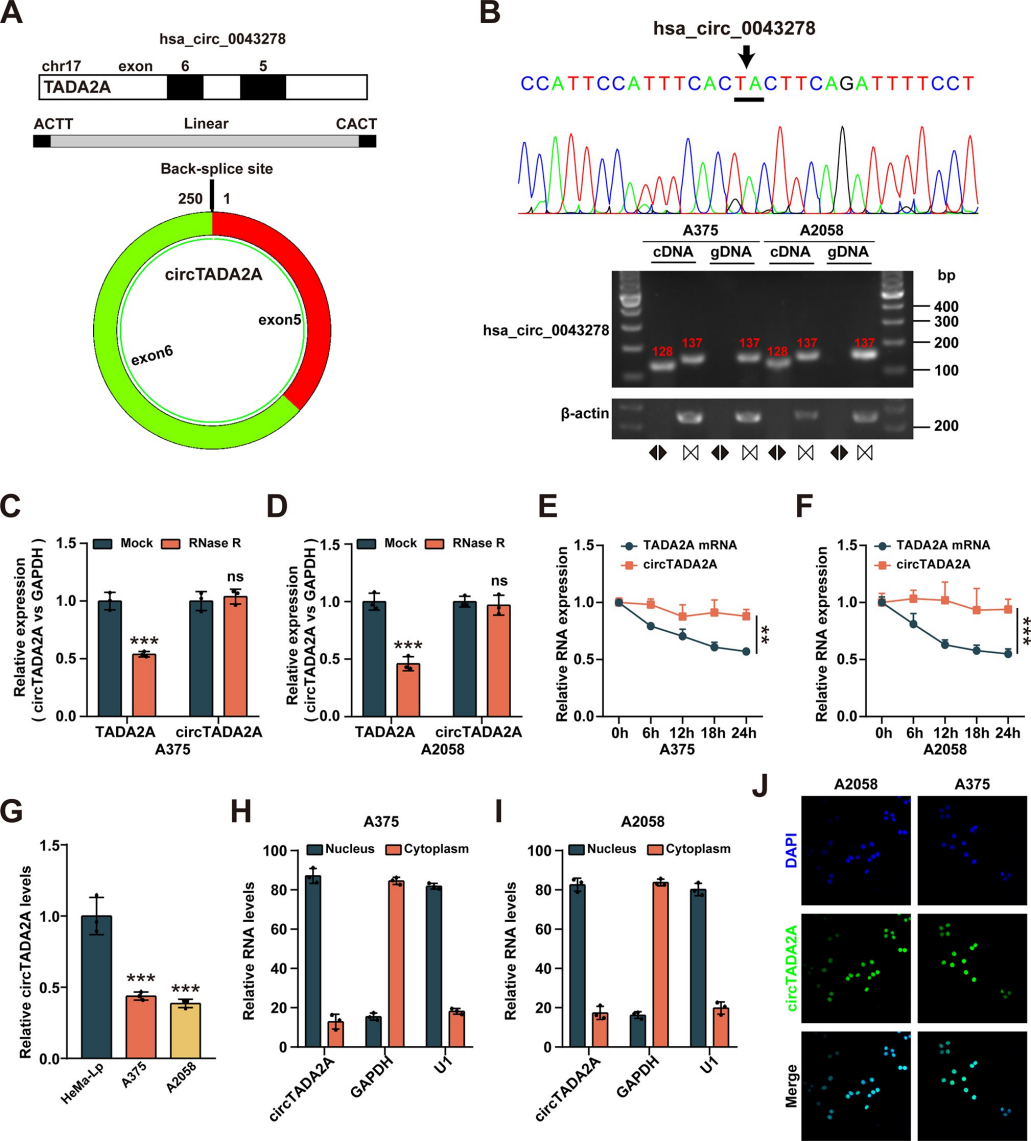
Characterization of circTADA2A in melanoma cells. **(A)** A schematic diagram showing that circTADA2A was formed by the back-splicing of linear TADA2A between the 5th and 6th exons. **(B)** The circTADA2A junction site was identified by Sanger sequencing. circTADA2A and its linear counterpart TADA2A in complementary DNA (cDNA) and genomic DNA (gDNA) was analyzed by qRT-PCR assays. **(C, D)** CircTADA2A and TADA2A mRNA were detected by qRT-PCR assays, RNA samples were treated with RNase R or mock treated without the enzyme. **(E, F)** qRT-PCR analysis for the abundance of circTADA2A and TADA2A in melanoma cells treated with Actinomycin D at the indicated time point. **(G)** qRT-PCR analysis for the abundance of circTADA2A in normal melanocytes cell line HeMa-Lp and melanoma cell line A375 and A2058. **(H, I)** Subcellular qRT-PCR analysis showing that circTADA2A was mainly localized in the nucleus. GAPDH and U1 were applied as positive controls in the cytoplasm and nucleus, respectively. **(J)** RNA fluorescence in situ hybridization analysis revealed the subcellular localization of circTADA2A.

The genomic structure showed that hsa_circ_0043278 (annotated as circTADA2A), which is composed of the 5 and 6 exons from the Transcriptional Adaptor 2A (TADA2A) gene (**Fig 1A**). The putative back-spliced junction fragment of circTADA2A was confirmed by Sanger sequencing, and further validated by PCR amplification with divergent primers from complementary DNA (cDNA), but not from genomic DNA in melanoma cell lines (**Fig 1B**). Moreover, the linear form of TADA2A was significantly reduced under RNase R treatment compared with mock, while circTADA2A was significantly resistant to RNase R in A375 and A2058 cells (**Fig 1C and 1D**). According to the RNA stability assay, circular transcript circTADA2A was much more stable than the linear mRNA transcript in melanoma cells under treatment with transcription inhibitor (actinomycin D) (**Fig 1E and 1F**). Subsequently, two melanoma cell lines (A375 and A2058) were selected for further investigations the circTADA2A expression levels. As shown in **Fig 1G**, the expression of circTADA2A was significantly downregulated in melanoma cells compared with HeMa-Lp cell. In order to further examined the subcellular localization of circTADA2A, we next performed the cellular RNA fractionation and FISH assays in the melanoma cells. We found that CircTADA2A was primarily distributed in the nucleus of melanoma cells (**Fig 1H–1J**). All these results indicated that circTADA2A was a bona fide circRNAs, which downregulated in melanoma cells.

### CircTADA2A serves as a tumor suppressor in melanoma

**Fig 2:**
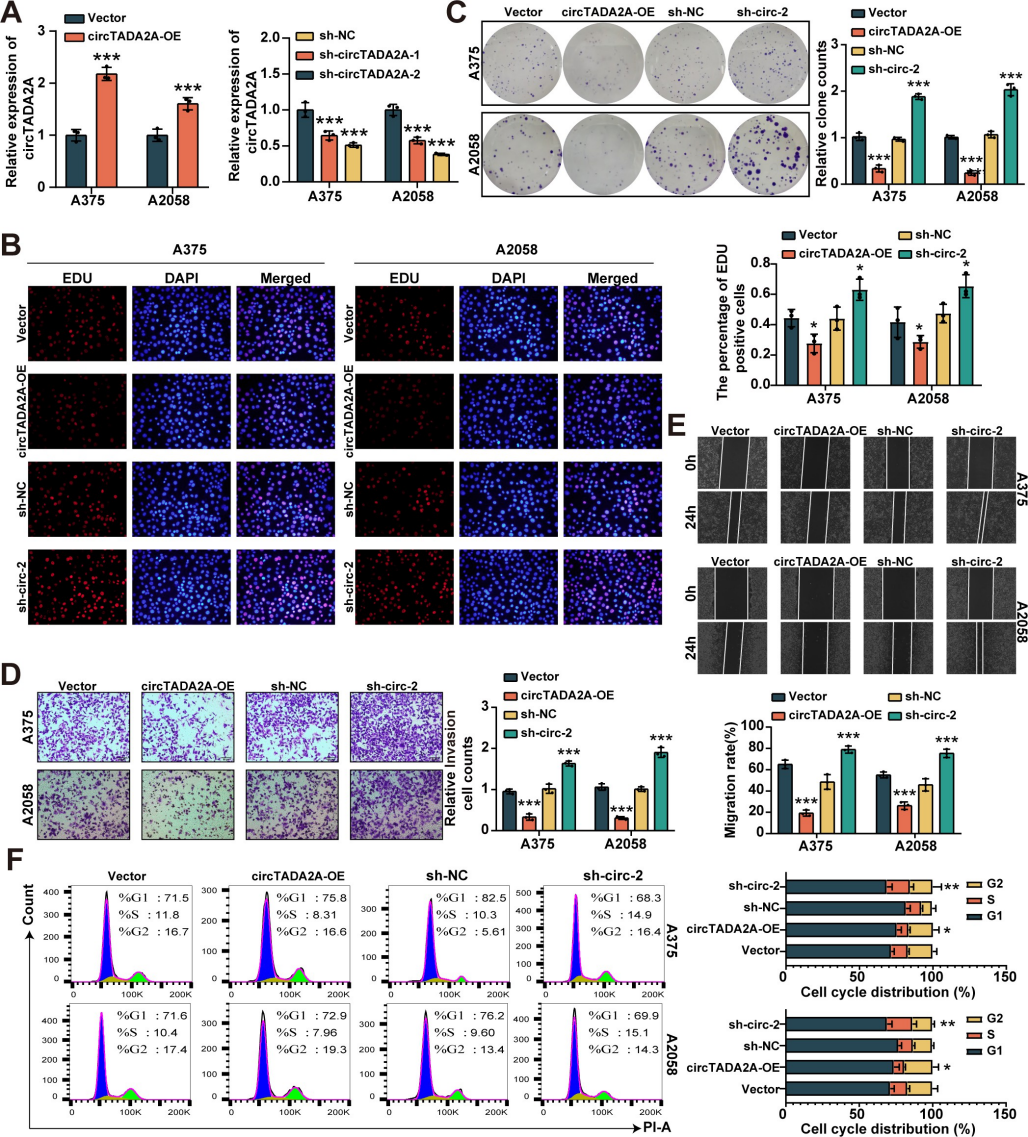
CircTADA2A suppresses proliferation, migration and invasion of melanoma cells. **(A)** The expression of circTADA2A was determined by qRT-PCR in circTADA2A overexpression or knockdown melanoma cells. **(B, C)** The effect of circTADA2A on melanoma cell proliferation was determined by EdU assay (B), and colony formation (C). **(D, E)** The effect of circTADA2A on melanoma cell migration and invasion was determined by transwell assay (D), and wound healing assay (E). **(F)** The effect of circTADA2A on melanoma cell cycle was determined by flow cytometry.

In order to comprehend the biological function of circTADA2A in the development of melanoma, we first constructed the circTADA2A overexpression system by using Lv-circRNA and the circTADA2A knockdown system by using sh-circRNA in melanoma cells. As shown in **Fig 2A**, circTADA2A was perfectly upregulated in A375 and A2058 cells by circTADA2A-overexpressing plasmid, and the transfection of sh-circTADA2A-1 or sh-circTADA2A-2 led to a distinct suppression in circTADA2A expression in A375 and A2058 cells. Besides, the sh-circTADA2A-2 had the strongest inhibitory effect on the expression of circTADA2A, so sh-circTADA2A-2 was selected for the follow-up experiments. Then, the cell proliferation capacity was analyzed by EDU incorporation and colony formation assays, and the results revealed that overexpression of circTADA2A decreased EDU-positive cell counts (**Fig 2B**) and clone forming numbers (**Fig 2C**) in melanoma compared with negative controls. On the contrary, circTADA2A knockdown showed the opposite result. Moreover, wound-healing and transwell assays were utilized to investigate melanoma cell migration. As shown in **Fig 2D** and **2E**, overexpression of circTADA2A could significantly decreased the numbers of migrated melanoma cells. However, with sh-circTADA2A, the migration ability of melanoma cells was promoted. Finally, the percentages of melanoma cells in different phases of cell cycle were analyzed by flow cytometry. We found that cell cycle in G1/S transition was remarkably halted by circTADA2A overexpression, while it was promoted by knockdown of circTADA2A (**Fig 2F**). Overall, these results demonstrated that circTADA2A repressed the progression of melanoma in vitro.

### CircTADA2A interacts with CNBP protein in melanoma cells

**Fig 3:**
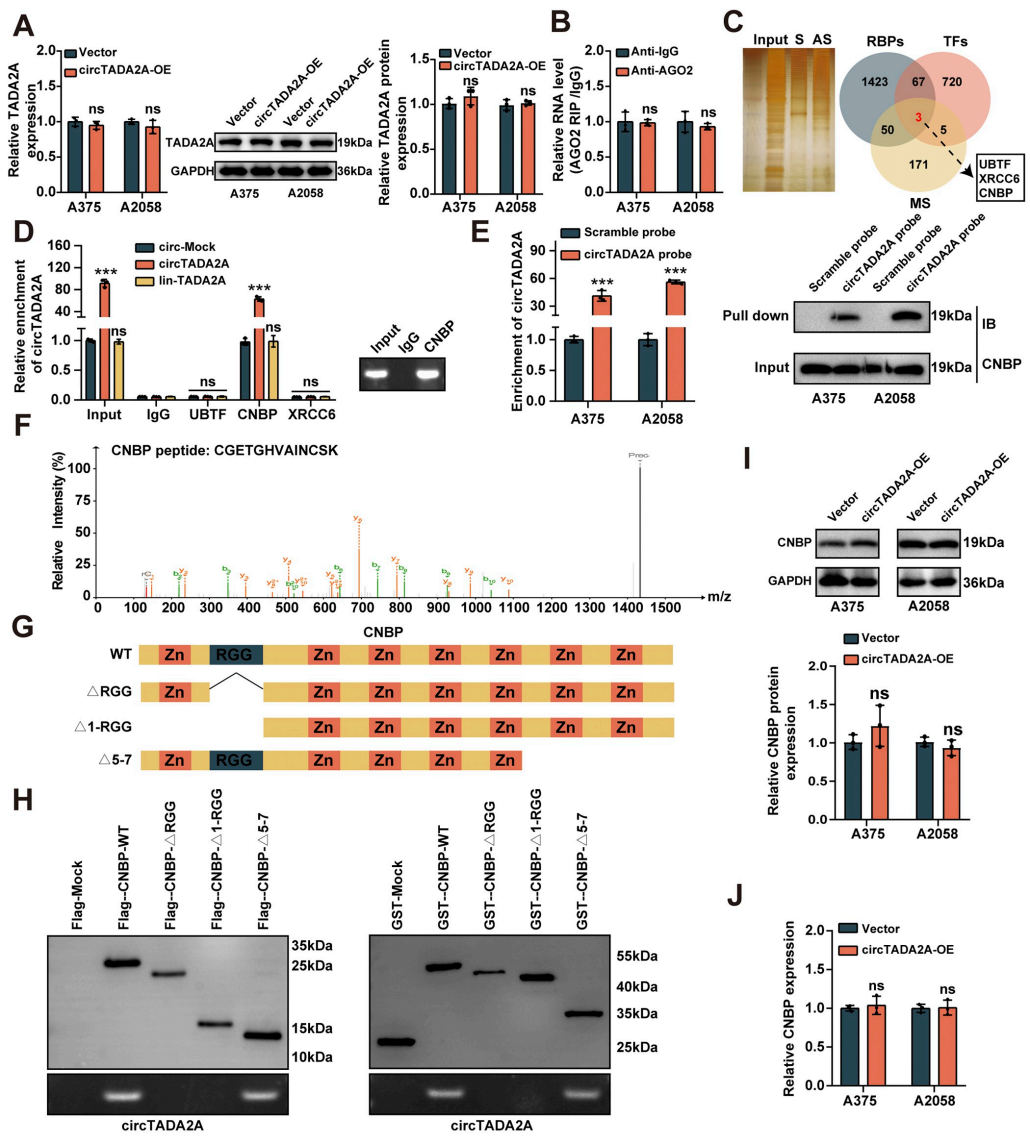
CircTADA2A interacts with CNBP protein in melanoma. **(A)** qRT-PCR analysis of TADA2A mRNA expression in melanoma cells with circTADA2A overexpression. **(B)** RIP assay revealed that the enrichment of circTADA2A on Ago2 relative to IgG. **(C)** SDS-PAGE separation and silver staining of the immunoprecipitated proteins obtained from A375 cells (left) by sense and anti-sense probe of circTADA2A. The proteins pulled down by probes were overlapped with established RBPs and TFs (right). **(D)** RIP and qRT-PCR assays showing the relative interaction between circTADA2A and three proteins in melanoma cells stably transfected with empty vector (circ-Mock), circTADA2A, or linear circTADA2A (lin-TADA2A), with normalization to input of cells transfected with circ-Mock. **(E)** RNA pulldown assay indicating the direct interaction between circTADA2A and CNBP in melanoma cells. **(F)** MS assay depicting the identified CNBP peptides pulled down by circTADA2A. **(G)** Schematic diagram revealing the domains of CNBP truncations. **(H)** In vitro binding assay showing the enriched circTADA2A levels detected by qRT-PCR after incubation with full-length or truncations of Flag-tagged or GST-tagged recombinant CNBP protein validated by western blot. **(I, J)** Western blot assays (I) and qRT-PCT (J) showing the protein levels of CNBP in A375 and A2058 cells stably transfected with mock or circTADA2A.

According to the result of qRT-PCR, we found that exogenous circTADA2A overexpression had no significant effect on the maternal TADA2A mRNA level in both cell lines (**Fig 3A**). Moreover, the RIP assay indicated that circTADA2A cannot be pulled down by AGO2 protein, suggesting that circTADA2A does not function through ceRNA mechanism (**Fig 3B**). Next, in order to explore the cancer-inhibiting mechanism of circTADA2A, we performed proteomic analysis of circTADA2A-associated protein complex in A375 cells by biotin-labeled circular or linear RNA pull-down assay. Mass spectrometry (MS) assay revealed 229 differential proteins between circular and linear circTADA2A pull-down groups, and overlapping analysis with established RBPs and transcription factors indicated three potential circTADA2A-interacting partners (**Fig 3C**). Importantly, RIP assay indicated that circTADA2A could bind to CNBP, rather than UBTF, or XRCC6 (**Fig 3D**). Moreover, the RNA pull-down assay also confirmed CNBP was the partner of circTADA2A (**Fig 3E**). The identified peptides of CNBP from MS assay were shown in **Fig 3F**. In vitro binding assay indicated that Arg-Gly-Gly (RGG) box domain (22–42 amino acids), but not other domains, of Flag-tagged or GST-tagged CNBP protein was crucial for its interaction with circTADA2A (**Fig 3G and 3H**). Notably, the protein and mRNA levels of CNBP were not change in A375 and A2058 cells with stable overexpression of circTADA2A (**Fig 3I and 3J**). Taken together, these results confirmed that circTADA2A could interact with CNBP in melanoma cells.

### SLC38A1 was a downstream target of circTADA2A/CNBP in melanoma

**Fig 4:**
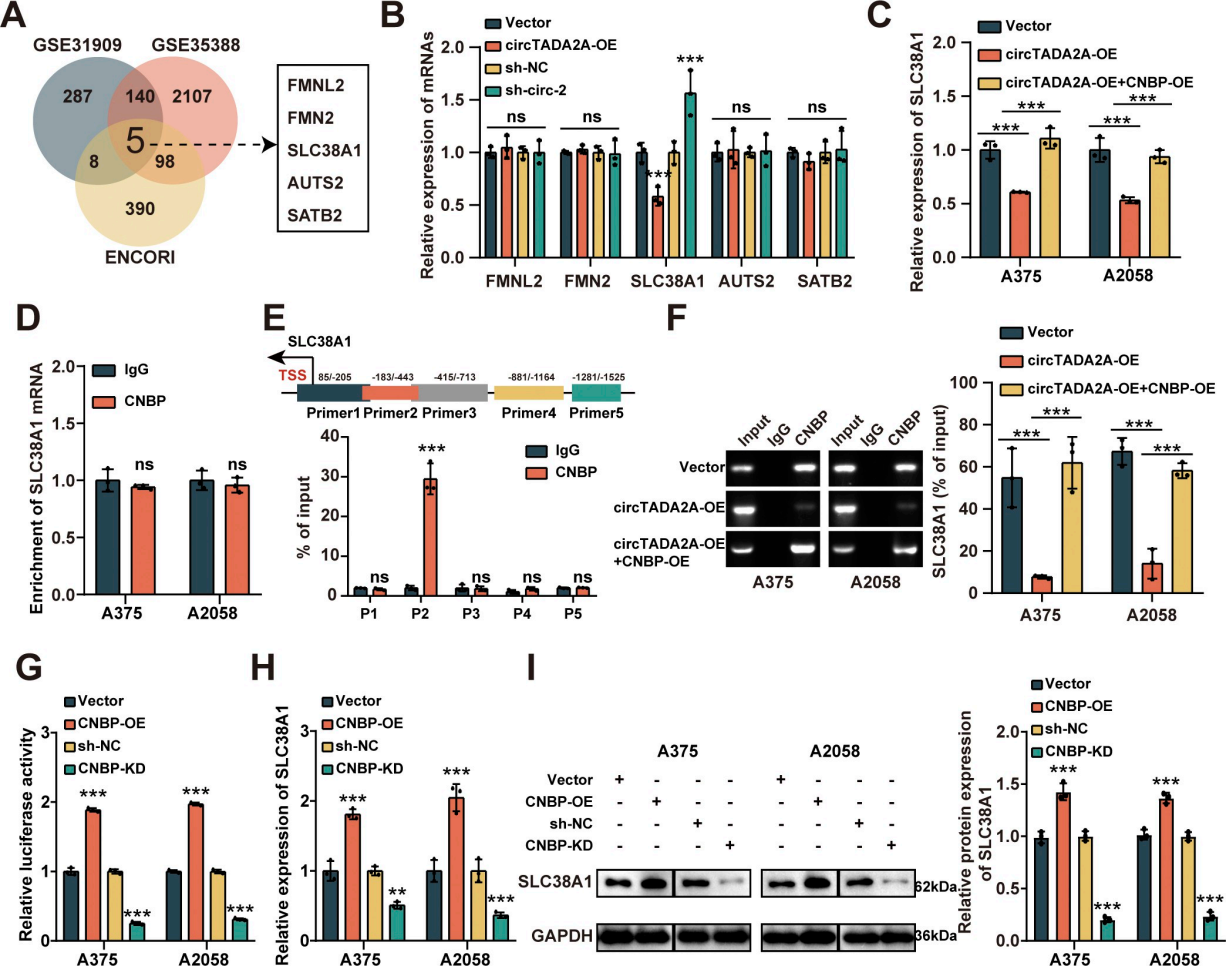
SLC38A1 was a direct downstream target of CNBP in melanoma. **(A)** The differentially expressed genes from melanoma datasets (GSE31909 and GSE35388) were overlapped with predicted targets of CNBP from ENCORI database. (**B)** The abundance of five potential target mRNAs in melanoma cells with circTADA2A overexpression or knockdown was analyzed by qRT-PCR assay. **(C)** qRT-PCR assay showing the expression of SLC38A1 in melanoma cells stably transfected with mock or circTADA2A, and those cotransfected CNBP. **(D)** RIP assay was used to evaluate interaction between CNBP and SLC38A1 mRNA. **(E)** CHIP assay with primer sets indicating the interaction between CNBP and SLC38A1 promoter in melanoma cells. (**F)** CHIP assay indicating the interaction between CNBP and SLC38A1 promoter in melanoma cells stably transfected with circ-Mock, circTADA2A, and those cotransfected CNBP. (**G)** Dual-luciferase reporting experiment was applied to evaluate the binding strength between CNBP and SLC38A1 promoter with Mock, CNBP-OE or CNBP-KD vectors. (**H)** qRT-PCR assay showing the abundance of SLC38A1 in melanoma cells stably transfected with mock, CNBP, sh-NC or sh-CNBP. (**I)** Western blot assay showing the expression of SLC38A1 in melanoma cells stably transfected with mock, CNBP, sh-NC or sh-CNBP.

Then, a series of experiments were conducted to investigate the underlying mechanism of circTADA2A/CNBP and its down-stream factors. Firstly, differentially expressed genes from Gene Expression Omnibus (GEO) datasets (GSE31909, GSE35388) were overlapped with predicted targets from ENCORI database, and a total number of five overlapped potential genes were identified, those were FMNL2, FMN2, AUTS2, SATB2 and SLC38A1 (**Fig 4A**). The results of qRT-PCR showed that circTADA2A overexpression or knockdown in melanoma cells could inhibit or enhance SLC38A1 mRNA expression, but had no effect on the expression of FMNL2, FMN2, AUTS2 and SATB2 mRNA (**Fig 4B**). In addition, stable transfection of CNBP into A375 and A2058 cells reversed the decreased expression of SLC38A1 caused by circTADA2A overexpression (**Fig 4C**). RIP assay showed that SLC38A1 mRNA could not be enriched by CNBP in A375 and A2058 cells (**Fig 4D**), indicating that CNBP does not function by acting as an RNA-binding protein. We further explored whether CNBP functions by acting as a transcription factor and the CHIP results showed that CNBP mainly interacts with the region amplified the SLC38A1 primer 2 (-530/-380) (**Fig 4E**). Moreover, overexpression of circTADA2A decreased the SLC38A1 promoter enrichment by CNBP, while this phenomenon could be reversed by enhanced expression of CNBP (**Fig 4F**). Transfection of CNBP enhanced the luciferase reporter activity, while CNBP knockdown reduced the luciferase reporter activity in A375 and A2058 cells (**Fig 4G**). Furthermore, the mRNA and protein levels of SLC38A1 were increased or decreased in A375 and A2058 cells with stable overexpression or knockdown of CNBP, respectively (**Fig 4H and 4I**). These data confirmed that SLC38A1 was a direct downstream target of circTADA2A/CNBP in melanoma cells.

### CircTADA2A suppresses SLC38A1 expression, growth, and invasion of melanoma cells via repressing CNBP transactivation

**Fig 5:**
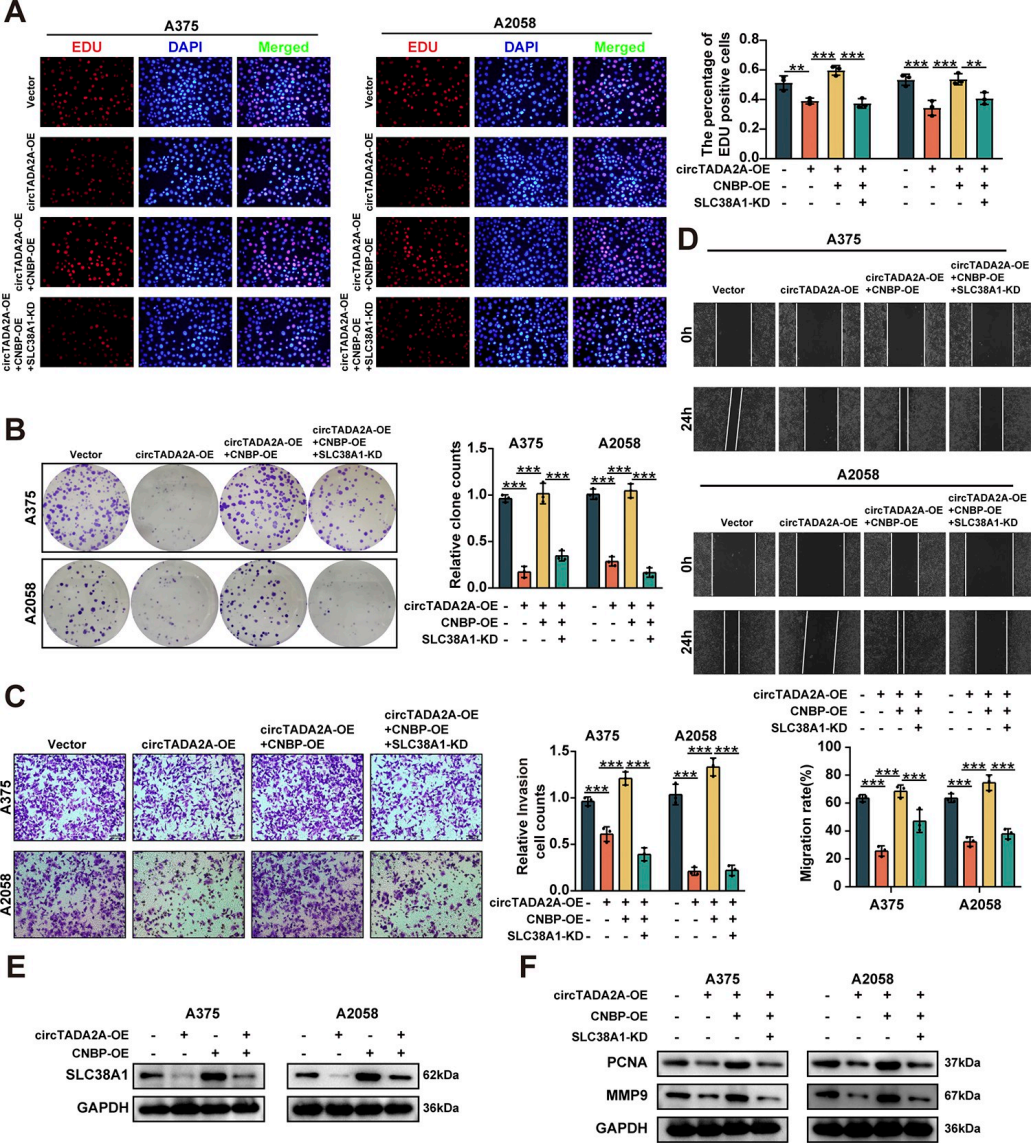
CircTADA2A/CNBP suppresses the progression of melanoma cells via repressing SLC38A1 expression. **(A, B)** EdU assay (A), and colony formation (B) assay indicating the cell proliferation of A375 and A2058 cells stably transfected with mock, circTADA2A, circTADA2A co-transfected with CNBP, or circTADA2A co-transfected with CNBP and sh-SLC38A1. **(C, D)** Transwell (C), and wound healing (D) assay indicating the cell migration and invasion of A375 and A2058 cells stably transfected with mock, circTADA2A, circTADA2A co-transfected with CNBP, or circTADA2A co-transfected with CNBP and sh-SLC38A1. **(E)** Western blot assay showing the expression of CNBP and SLC38A1 in melanoma cells stably transfected with circ-mock, circTADA2A, mock or CNBP. **(F)** Western blot assay showing the expression of PCNA and MMP-9 in melanoma cells stably transfected with mock, circTADA2A, circTADA2A co-transfected with CNBP, or circTADA2A co-transfected with CNBP and sh-SLC38A1.

In order to further investigate the interplay effects between circTADA2A and CNBP in regulating SLC38A1 expression and melanoma progression, A375 and A2058 cells were co-transfected with circTADA2A overexpression vector, CNBP overexpression vector and SLC38A1 knockdown vector. Transfection of circTADA2A overexpression vector suppressed the proliferation, migration and invasion of melanoma cells. And co-transfection of circTADA2A overexpression vector and CNBP overexpression vector in A375 and A2058 cells rescued cell proliferation, migration and invasion, while these effects could be blocked by SLC38A1 knockdown (**Fig 5A–5D**). Moreover, stable overexpression of circTADA2A attenuated the protein expression levels of SLC38A1 in melanoma cells, which were blocked by CNBP overexpression (**Fig 5E**). The decreased expression of PCNA and MMP-9 induced by circTADA2A overexpression was significantly blocked following CNBP overexpression, whereas CNBP-enhanced PCNA and MMP-9 expression was completely reversed by SLC38A1 knockdown (**Fig 5F**). Taken together, these results showed that circTADA2A suppressed melanoma cell progression via repressing CNBP/ SLC38A1 pathway.

## Discussion

Melanoma is the most common aggressive malignant skin tumor, and its incidence is on the rise [1, 16]. CircRNAs are widely expressed in mammals and have been indicated to play critical regulatory roles in tumorigenesis and progression [5]. Herein, we uncovered a novel circTADA2A had significantly downregulate in melanoma cells and repressive effects on melanoma cell proliferation, invasion and metastasis in vitro. By function assay, we found that circTADA2A exhibit its inhibitory effects in cancer progression via interacting with CNBP proteins to regulate SLC38A1 transcription.

CircRNAs are a type of stable ncRNAs characterized by tissue-specific expression and high evolutionary conservation [17]. An increasing number of studies have implicated that circRNA play pivotal roles in tumor initiation and progression [18], and might function as diagnosis markers and therapeutic molecules in cancer [19]. The most studied mechanism is that circRNA serve as competing endogenous RNAs (ceRNAs) to sponge microRNA (miRNA), thereby relieving the inhibition effect of miRNA on its target [20]. In addition, circRNAs is also involved in cancer progression through interaction with RBPs to block or enhance protein function [21]. CircTADA2A is identified from exons 5 and 6 of the TADA2A gene [11], and plays an important role in several tumor. For example, circTADA2A has been shown to promote the progression of osteosarcoma by sponging miR-203a-3p [11]. CircTADA2A was shown to regulate Kruppel like factor 14 (KLF14) via binding to miR-374a-3p, and then suppress colorectal cancer progression [13]. Xu and colleagues have reported that circTADA2As repress breast cancer progression and metastasis by targeting miR-203a-3p/SOCS3 axis [14]. However, previous studies have mainly focused on the regulation role of circTADA2A as a ceRNA. Here, we determined that circTADA2A is downregulated in melanoma and effectively inhibits the progression of gastric melanoma cells. Most interestingly, circTADA2A directly interacted with CNBP, and acted as an inhibitor to restrain the binding of CNBP to its downstream proteins, resulting in repression of melanoma cell progression.

CNBP is a highly conserved zinc-finger protein with a broad sequence specificity [22]. Moreover, CNBP has been proposed to be involved in a variety of cellular functions, including transcription and translation [23, 24].

CNBP has been identified as a key transcriptional regulator that promotes immune response by maintaining interleukin-6 expression [25]. CNBP was shown to control transcription of c-MYC and KRAS oncogenes in human cancer by unfolding DNA G-quadruplex structures [26]. Moreover, CNBP can act as a RBP that is involved in tumor progression. For example, circFMN2 contributes to sorafenib resistance in hepatocellular carcinoma via upregulation of CNBP by restraining ubiquitination [27]. CircHuR inhibits gastric cancer progression via inhibiting CNBP-facilitated HuR expression [9]. In the present study, we found that CNBP regulates the expression of SLC38A1 as a transcription factor rather than an RNA-binding protein. Meanwhile, circTADA2A competitively binds CNBP to affect the regulatory effect of CNBP on SLC38A1 transcription. Notably, our results demonstrated that circTADA2A can bind CNBP, but does not regulate the expression of CNBP.

The circTADA2A/CNBP complex is likely to be located in the nucleus and may be required to regulate the transcription of genes involved in tumor progression. It is appealing to suspect that the circTADA2A/CNBP might also play essential roles in melanoma. Next, we identified SLC38A1 as the downstream target of circTADA2A/CNBP through gain- and loss-of-function assays. Pervious study has showed that SLC38A1 was highly expressed in hepatocellular carcinoma tissues, and associated with a poor prognosis [28]. Importantly, the expression of SLC38A1 is significantly up-regulated in human melanoma tissue. Knockdown SLC38A1 expression reduce the proliferation rate of melanoma cells [29]. Consistent with this finding, we found that circTADA2A/CNBP represses the progression of melanoma by inhibiting the SLC38A1expression.

In summary, we identified circTADA2A as a significantly downregulated circRNA in melanoma. CircTADA2A inhibited melanoma growth and metastasis by interacting with CNBP protein to modulate gene transcription of SLC38A1. These findings may have a fundamental impact on melanoma research, and may provide new therapeutic targets for melanoma treatment.

## Supporting information

S1 Raw images (PDF)

S1 Data (XLS)

S1 Raw data (TIF)

S2 Raw data (TIF)

S3 Raw data (TIF)
