# Supplementary figures and images for: circTADA2A inhibited SLC38A1 expression and suppresses melanoma progression through the prevention of CNBP trans-activation
Source: PLoS One. 2024 Apr 18;19(4):e0301356. doi: 10.1371/journal.pone.0301356 (PMC11025954; doi:10.1371/journal.pone.0301356)

**Fig. 3**

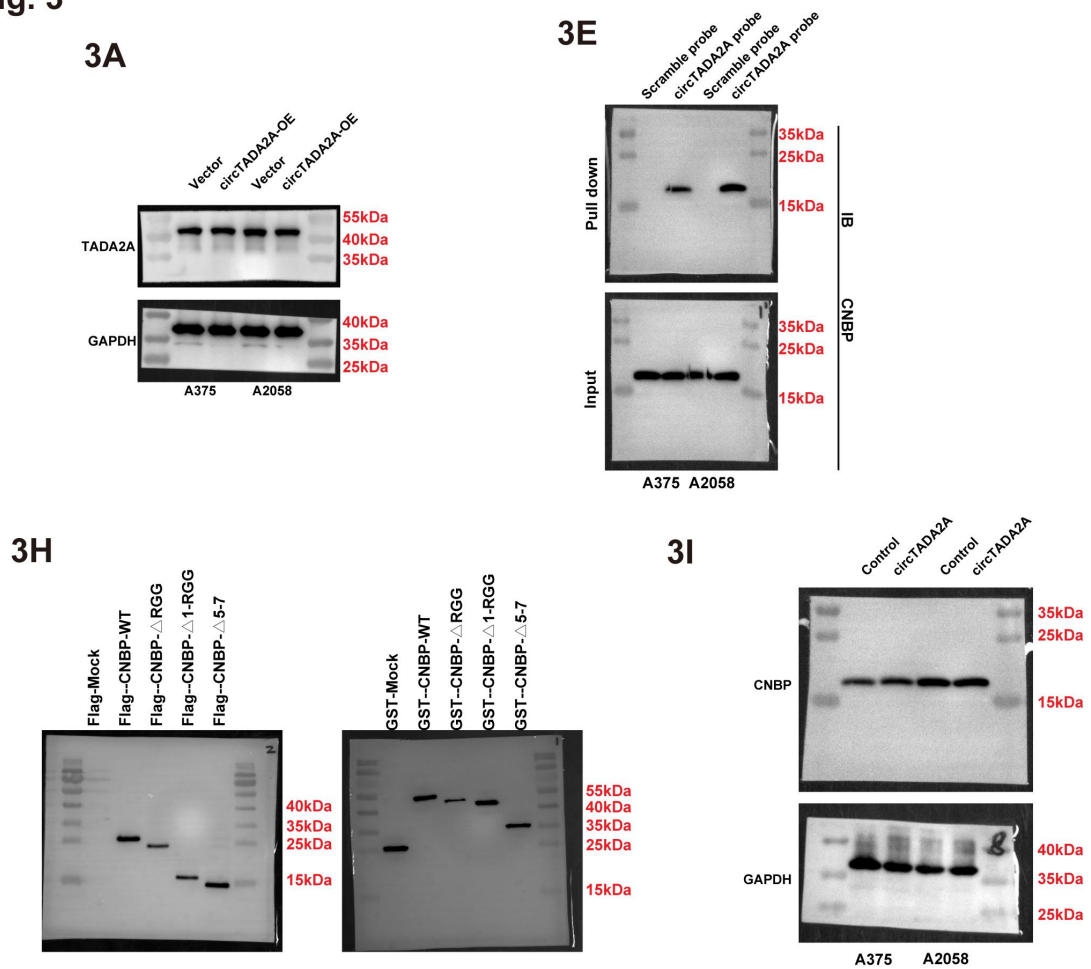

Fig. 4  
4l

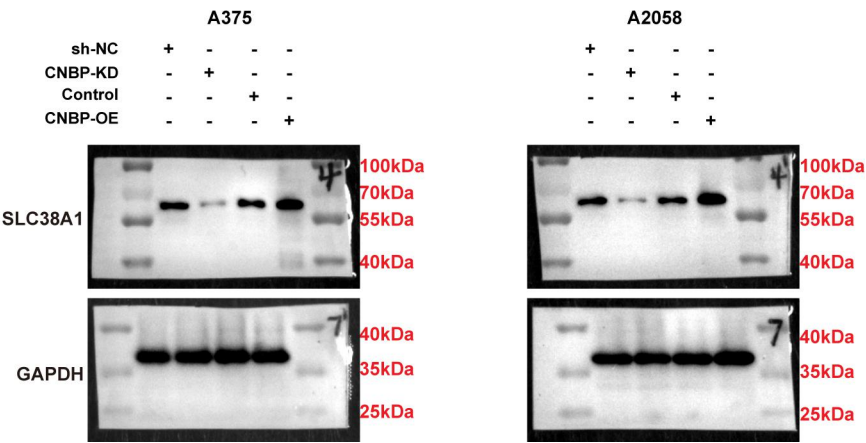

Fig. 5

5E

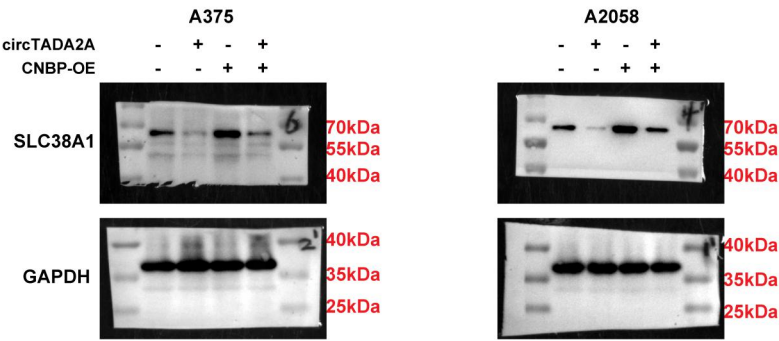

5F

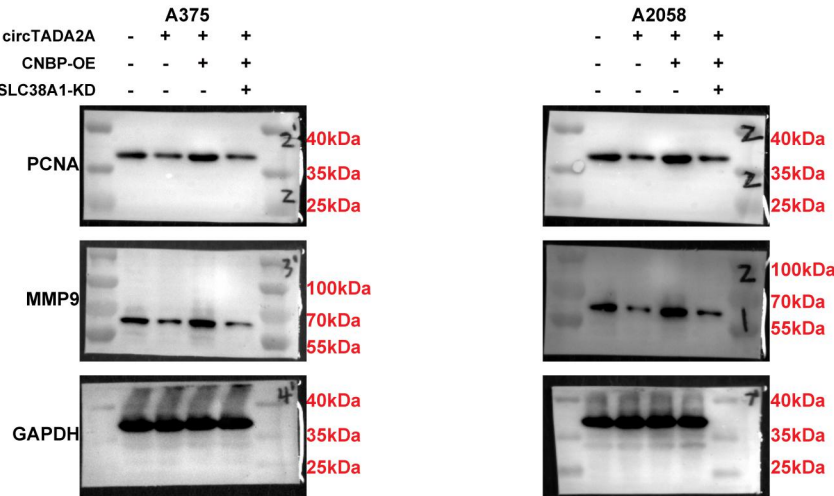

Supplement: S1 Raw images — (PDF) [file pone.0301356.s001.pdf]

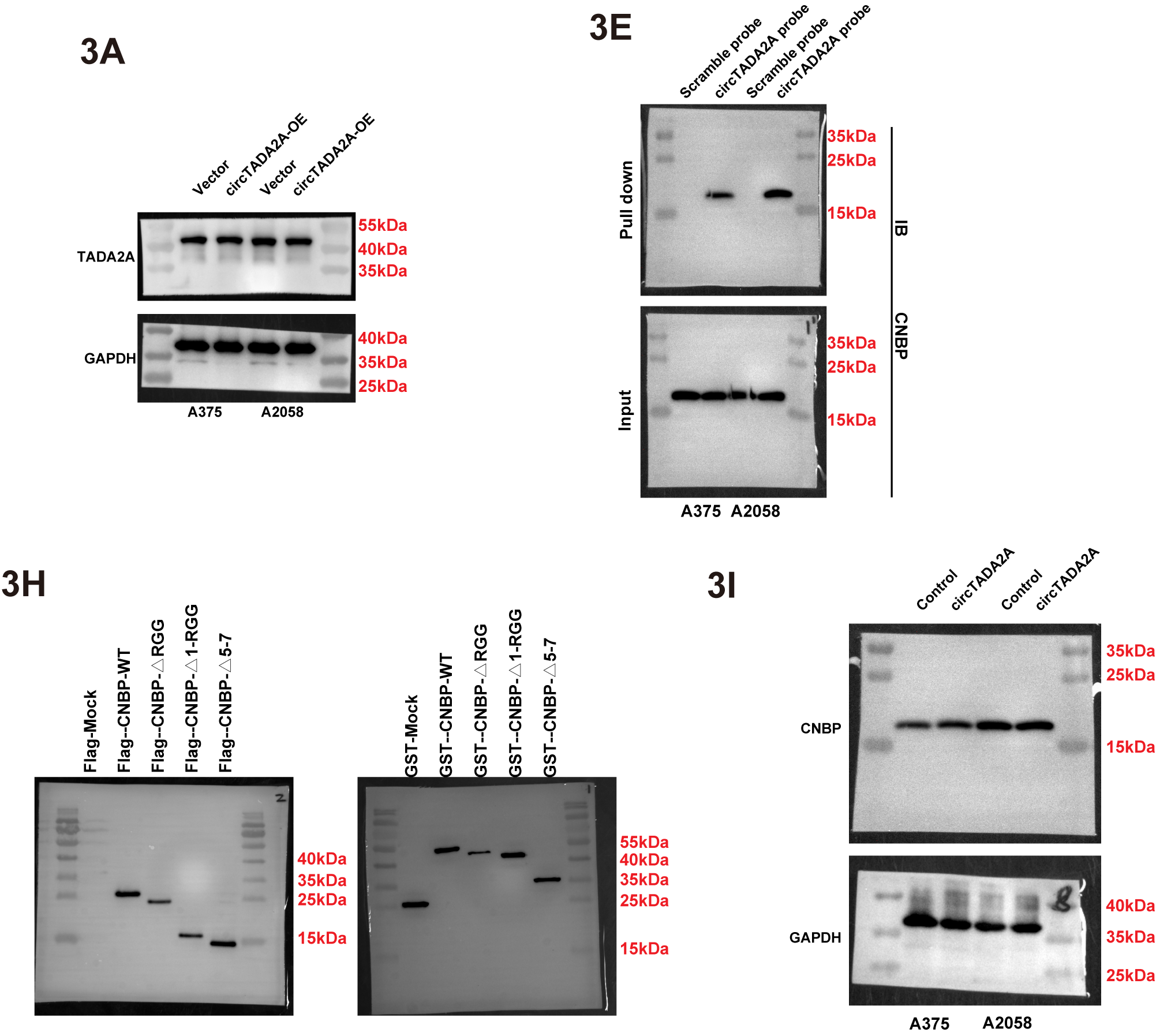

Supplement: S1 Raw data — (TIF) [file pone.0301356.s003.tif]

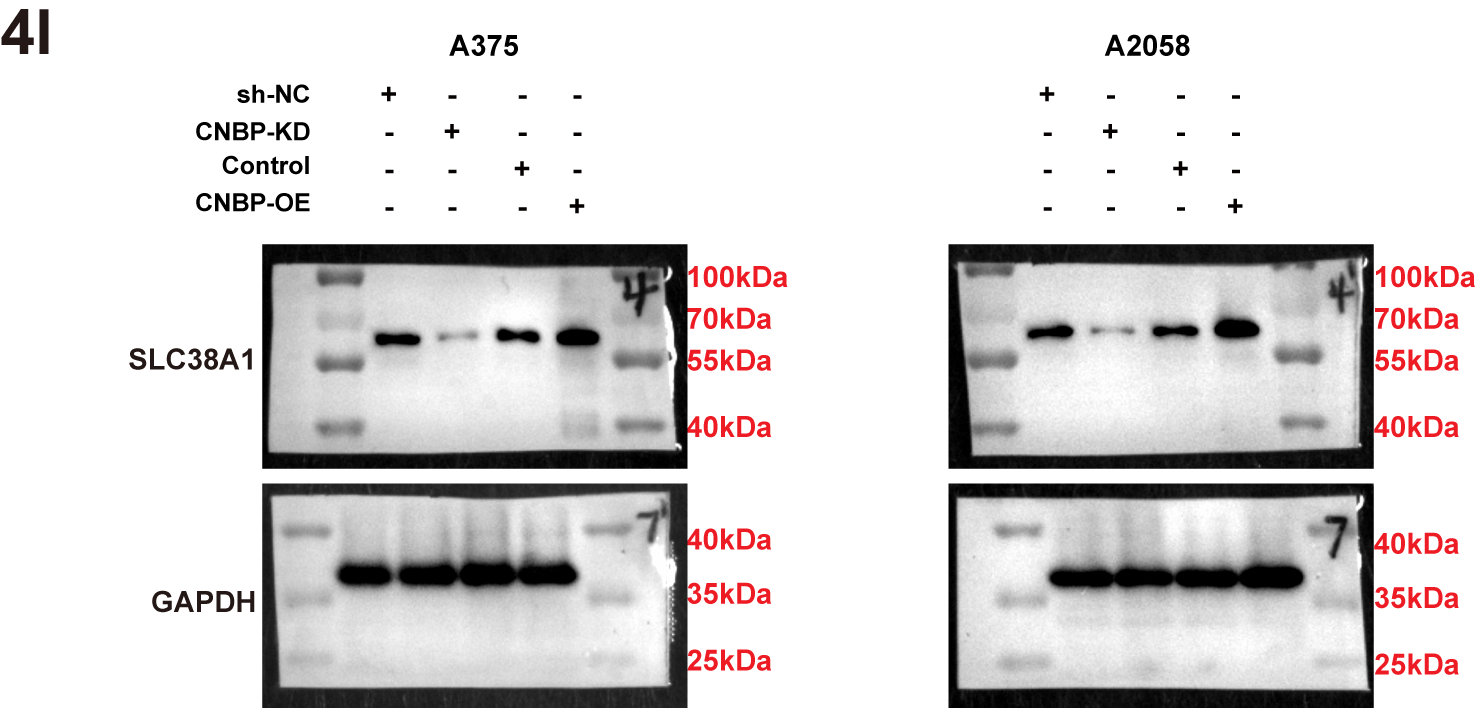

Supplement: S2 Raw data — (TIF) [file pone.0301356.s004.tif]

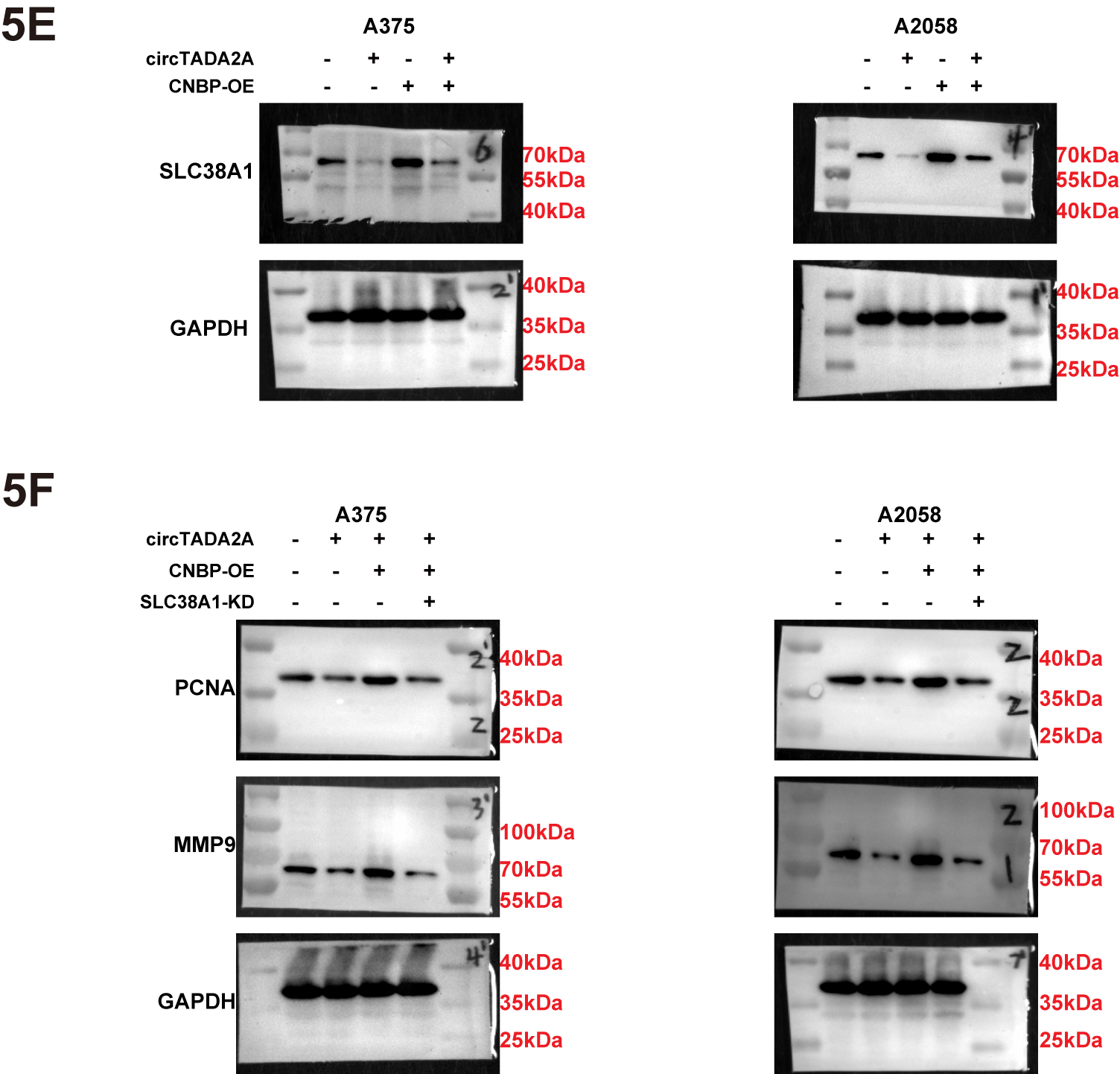

Supplement: S3 Raw data — (TIF) [file pone.0301356.s005.tif]
